# New Approach of High Sensitivity Techniques Using Collective Detection Method with Multiple GNSS Receivers

**DOI:** 10.3390/s18113690

**Published:** 2018-10-30

**Authors:** Maherizo Andrianarison, René Landry

**Affiliations:** LASSENA Laboratory, Department of Electrical Engineering, École de Technologie Supérieure, Montréal, QC H3C 1K3, Canada; renejr.landry@etsmtl.ca

**Keywords:** collective detection, high sensitivity, cooperative positioning, cognitive GNSS receivers, weak signals, challenging environments

## Abstract

The Collective Detection (CD) technique is a promising approach to meet the requirements for signal acquisition in GNSS-harsh environments. The CD approach has been proposed because of its potential to operate as both a direct positioning method and a high-sensitivity acquisition method. This paper is dedicated to the development of a new CD architecture for processing satellite signals in challenging environments. It proposes the best signal acquisition method used according to the reception conditions of the different receivers that can assist the user in difficulty. Knowing that the CD approach is beneficial in the case where the maximum of satellite signals can be combined, the proposed approach consists in choosing the best receiver(s) from several connected receivers to serve as a reference station, as smart cooperative navigation concept. New metrics of the CD with optimal weighting of visible satellites are exploited. Analysis of optimization method in order to use better satellites according to some defined parameters (elevation, $C/N_{0}$, and GDOP) were carried out. Real GPS L1 C/A signals are exploited to analyze the efficiency of the proposed approach. A comparison of the results through the accumulation of some good satellites among all visible satellites have shown the effectiveness of this method.

## 1. Introduction

In recent years, the interest in positioning and navigation has become increasingly important, as evidenced by systems and services developed using this technology. The use of GNSS receivers has increased considerably with the integration of GNSS chips in cellular phones and mobile devices that are used for potential applications in several domains, including location-based services (LBS) and intelligent transportation systems (ITS). Constantly increasing the number of GNSS signals and the diversity of navigation satellites is an important advantage, because it can improve the overall performance of the signal processing in a multi-constellation multi-frequency hybrid architecture. Traditional satellite navigation receivers must therefore be improved in order to allow the reception of all GNSS signals (current and future). These receivers must be designed to acquire as many satellite signals as possible from different constellations.

The diversity and redundancy of GNSS measurements will further open the door to new developments and applications that are impossible to achieve with current receivers, which are designed for outdoor environments. Increasing the number of signals and the diversity of GNSS satellites provides significant benefits in many areas of application because it can improve the sensitivity performance of the signal processing in a hybrid architecture combining all GNSS signals.

Overcoming the problem of positioning in hostile environments is best achieved using high sensitivity (HS) and robust receivers. Several techniques have been proposed to deal with this problem of receiving weak signals in urban environments. For indoor environments, many technologies have been proposed for localization purposes, because GNSS signals cannot be received with a sufficiently high signal-to-noise ratio [1]. Moreover, most techniques require a dedicated physical infrastructure.

The Collective Detection (CD) technique is considered as a promising approach to perform GNSS signal reception and processing in harsh environments. It is a powerful method for acquiring highly attenuated satellite signals in constrained environments as it can process all visible satellites collectively taking advantage of the spatial correlation between GNSS signals as a vector acquisition scheme. The major advantage of CD as a collective acquisition method lies in its ability to use stronger signals to facilitate the acquisition of weaker ones and acquisition in harsh environments could be used directly for positioning without the need for standard tracking loops [2,3,4]. Indeed, the GNSS receiver antenna receives at least one strong signal which is then used to detect weak satellite signals in view, in a sort of multi-satellite collaborative processing. In the CD approach, the projection of the code phase onto the position/clock bias domain is done differentially with respect to the pseudorange measurements provided by a reference station with known position.

As the number of mobile devices in use increases, applying the CD approach becomes easier since devices that are capable of receiving good satellite signals can assist others in difficulty [5]. The main objective of this paper is to propose an architecture of a smart high sensitivity GNSS receiver based on the CD approach. 

**Figure 1:**
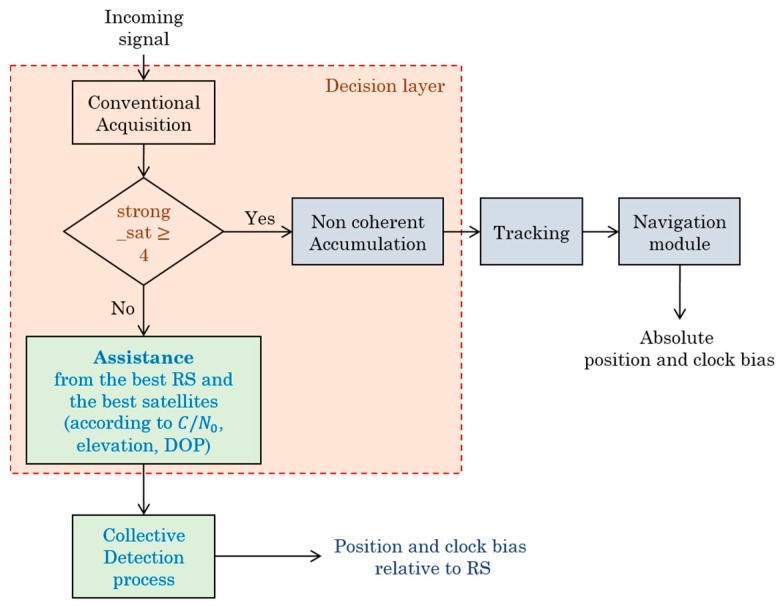
Smart functional architecture of a HS-SGR.

To design the architecture of an intelligent HS GNSS receiver, called as HS Smart GNSS Receiver (HS-SGR), we will begin by defining its basic operation according to the number of satellites detected as shown in Figure 1. If there are enough strong satellites (greater than or equal to 4) available to calculate the position of the receiver, a conventional sequential acquisition is carried out, otherwise, the CD process is used. Following this, the intelligence of the receiver must consider other parameters according to the acquisition method used to process the received satellite signals.

If there is no Reference Station (RS) receiver capable of assisting the user, here is an example of information that will be described in the HS-CGR cognitive layer:

- Depending on the type of receiver start-up (cold/warm/hot): use relevant information in the receiver or perform the normal process.
- According to $C/N_{0}$ level: use high sensitivity techniques (long coherent integration with non-coherent accumulation).
- Depending on the number of received GNSS signals: track more powerful signal by standard tracking or combine various available signals using collective detection.

When there are less than four satellites that can be detected individually, CD can still provide a coarse positioning solution with attenuated signals in a challenging environment. CD can be seen both as a high sensitivity and a direct positioning method because it provides a coarse estimation of the user position using direct navigation solution [3].

The CD approach is able to process weak GNSS signals, but is however, computationally intensive because of the important number of candidate points considered to search the position and the available assistance data. The computational load is even greater if the CD process is performed using the accumulation of all visible satellites which makes its practical implementation very difficult [6,7,8,9,10]. Thus, this paper proposes a method that consists in choosing the best surrounding receiver from several connected receivers to serve as a reference station to assist other receivers in difficulty, in a smart cooperative navigation set-up. In this proposal, various parameters will be taken into consideration when choosing the best reference receiver. Then, new metrics of the CD involving an optimal weighting of visible satellites will be exploited. An optimization method will be analyzed in order to ensure that the best satellites are used in the CD process according to certain defined parameters such as the elevation mask angle, the signal power ($C/N_{0}$), and the geometry of the satellite constellation (Geometric dilution of precision—GDOP).

In order to ensure a better sensitivity performance in the CD process, with the best receiver chosen from among several connected receivers, the Spectral Peak Location (SPL) delta corrected FFT technique, developed in [11], is used for Doppler estimation. It can be seen that this proposal provides certain advantages in terms of complexity and sensitivity performance, compared to other Doppler estimation techniques in the position and velocity domain as [12]. 

The rest of the paper is structured as follows: the CD principle is presented in Section 2. Then, Section 3 develops the proposed smart collaborative positioning using the CD approach (smart high sensitivity receiver). In Section 3, we present the influence of certain parameters in the CD process, the principle of exploitation of best satellites while presenting the new metrics of CD and the algorithm of smart CD using the best satellites. Results with real GNSS signals will be presented in Section 4 to show the efficiency of the proposed method. Finally, we conclude the paper with a discussion about the overall performance achieved with this proposal, the contribution of this work and the outstanding issues to be covered in future work.

## 2. Collective Detection Approach

### 2.1. Dependence on Assistance Data

The concept of CD is intended to complement an existing Assisted-GNSS (A-GNSS) and positioning method. In fact, Collective Detection is an A-GNSS approach for direct positioning in which all information from satellites in view are combined in order to enable rapid acquisition, i.e., to reduce the time to first fix (TTFF) and increase the sensitivity of the receiver. The assistance data allows the GNSS receiver to reduce the search space by providing assistance information [13].

It should be noted that for the application of the CD principle, apart from the RS position (for reducing the initial Mobile Station (MS) spatial uncertainty) and the ephemeris (for computation of the expected satellite position and velocity from azimuth and elevation angles), a new assistance parameter is required, which is the pseudorange measurements for all visible satellites as seen from the reference station. This is used in the code phase estimation of each candidate point in the position domain to estimate the user position. 

### 2.2. Working Principle of Collective Detection

**Figure 2:**
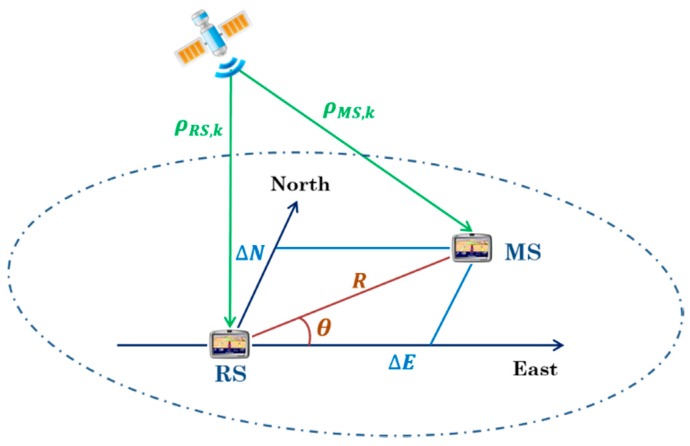
Mapping of the MS code delay search to position/clock bias and pseudorange domains.

The CD approach has been proposed because of its use as both a Direct Positioning method and a High-Sensitivity acquisition method. The direct positioning algorithms are based on a set of individual correlograms formed by code delay/Doppler for the satellites potentially visible. The direct positioning search is carried out in the position/clock bias domain with each potential position and clock bias coordinate is mapped to the code delay and Doppler space for each satellite. The mapping of the signal code delay to the position/clock bias domain of the user (MS) is done differentially with the respect to the pseudorange measurements provided by the reference station (RS), as shown in Figure 2.

The acquisition search grid is set in a space defined by 3D position coordinates (ΔN,ΔE,ΔD) which are the algebraic distances between the MS and the RS in North, East and Down directions, and the relative clock bias of the receiver to the reference station (ΔB).

The pseudorange seen by the MS for the satellite k can be calculated by [7]:

(1) $$\;\rho_{\mathrm{MS},k}=\rho_{\mathrm{RS},k}+\Delta \rho_{k}\;$$

where $\rho_{\mathrm{RS},k}$ is the pseudorange seen by the RS, and $\Delta \rho_{k}$ represents the difference in pseudorange calculated from the MS w.r.t the one measured by the RS for the satellite k. In CD, the uncertainty space is centered on the initial position and clock bias, and can be defined w.r.t. the accuracy of the initial knowledge. The range-offset at a position separated by (ΔN,ΔE,ΔD,ΔB) from the RS is expressed in terms of the position and the clock bias [2]:

(2) $$\;\Delta \rho_{k}\left(\Delta N,\Delta E,\Delta D,\Delta B\right)=-\cos\left(az_{k}\right)\cos\left(el_{k}\right)\Delta N\;\;-\sin\left(az_{k}\right)\cos\left(el_{k}\right)\Delta E\;\;+\sin\left(el_{k}\right)\Delta D\;+\;c\cdot \Delta B\;$$

where $az_{k}$ and $el_{k}$ are, respectively, the azimuth and elevation angles of satellite k as seen by the RS. The coordinates ΔN, ΔE and ΔD represent the 3D position displacement of the MS with respect to the RS in a North-East-Down (NED) local coordinate frame. The last term c·ΔB represents the pseudorange variation due to the clock bias of the MS, c being the speed of light.

In order to considerably decrease the total number of points to evaluate, the circular search area proposed in [7] is used in this paper. The local horizontal search is a polar Rho-theta referential instead of a North-East referential. In this way, ΔN and ΔE are expressed in terms of R and θ [7]:

(3) $$\;\{\begin{array}{c} \Delta N=R.\cos\left(\theta \right)\\ \Delta E=R.\sin\left(\theta \right) \end{array}$$

So, from (2) we obtain:

(4) $$\;\Delta \rho_{k}\left(R,\theta ,\Delta D,\Delta B\right)=-R\cos\left(el_{k}\right)\cos\left(az_{k}-\theta \right)+\sin\left(el_{k}\right)\Delta D+c.\Delta B\;$$

Thus, the estimated code delay for satellite k for a hypothetical location $\left(R_{i},\theta_{j},\Delta D_{m},\Delta B_{n}\right)$ is given by [7]:

(5) $$\;{\hat{\tau }}_{k}=\frac{{\left[\rho_{\mathrm{RS},k}+\Delta \rho_{k}\left(R_{i},\theta_{j},\Delta D_{m},\Delta B_{n}\right)\right]}_{c\cdot T_{code}}}{c\cdot T_{code}}\cdot N_{code}\;$$

where $T_{code}$ is the signal spreading code period (i.e., 1 ms for GPS L1 C/A code), $N_{code}$ is the number of code chips per period, and ${\left[.\right]}_{c\cdot T_{code}}$ represents the modulo $c\cdot T_{code}$ operation such that ${\hat{\tau }}_{k}\in \left[0,N_{code}-1\right]$ chip.

Then, the pseudorange can be converted to an equivalent estimated code phase ${\hat{\tau }}_{k}$, at a hypothetical location $R_{i},\theta_{j}$, a clock bias $\Delta B_{n}$, and the maximum code phase error, as [7]:

(6) $$\;{\hat{\tau }}_{k}=\frac{\Delta \rho_{k}\left(R_{i},\theta_{j},\Delta B_{n},\beta \right)}{c\cdot T_{code}}\cdot N_{code}\;$$

Then, the correlation output, which is the individual detection metric, is expressed as [7]:

(7) $$\;D_{ind}\left(R_{i},\theta_{j},\Delta B_{n},\beta \right)={\left|S\left({\hat{\tau }}_{k}\right)\right|}^{2}\;$$

Finally, the CD metric is obtained by the sum of individual detection metrics for all satellites in view as [7]:

(8) $$\;D_{\mathrm{CD}}\left(R_{i},\theta_{j},\Delta B_{n},\beta \right)=\underset{k}{\sum }D_{ind}\left({\hat{\tau }}_{k}\right)\;$$

Once the CD metric is performed for all candidate points, many approaches can be used to decide which set of values corresponds to the best estimation of the true MS position coordinates and clock bias. If the CD metric exceeds a pre-defined threshold, the GNSS signal could be detected. Thus, the code phase and Doppler frequency corresponding to the detected signal could be obtained.

The benefit of the CD application is shown in Equations (7) and (8). In fact, weak satellite signals may not be detectable in conventional GNSS receivers with only the individual correlator output value given by Equation (7). Nevertheless, the accumulation of all individual correlation values for each visible satellite can increase the receiver sensitivity using Equation (8) in which the summation operator represents the term “collective” in collective detection.

### 2.3. SPL Delta Corrected FFT Technique in CD

In this paper, the frequency estimation technique developed in [11] is adopted. It has been demonstrated that the SPL method of Doppler frequency estimation with delta correction used in CD makes it possible to increase the sensitivity of the receiver [8]. Thus, with some better satellite measurements in view, we can indeed have greater sensitivity. This is very interesting while we can limit the number of satellites used in the CD process but not all available visible satellites. The receiver complexity is thus reduced. Details of this proposal are detailed in [8]. By applying the Doppler estimation in the CD, the correlation values and the CD metrics are expressed as follows.

The delta-corrected Doppler is expressed as [11]:

(9) $$\;{\hat{f}}_{d}={\hat{f}}_{d_{k}}+{\hat{f}}_{\delta }\;$$

where ${\hat{f}}_{d_{k}}$ represents the Doppler frequency of the k-th satellite signal, and ${\hat{f}}_{\delta }$ represents the frequency correction term calculated by the fractional correction term, δ, as ${\hat{f}}_{\delta }=\hat{\delta }\cdot \delta f_{d}$.

Thus, the new delta-corrected coherent output is defined as [11]:

(10) $$\;S_{\delta }\left({\hat{\tau }}_{k},{\hat{f}}_{d}\right)=S\left({\hat{\tau }}_{k},{\hat{f}}_{d_{k}}+{\hat{f}}_{\delta }\right)\;\;S_{\delta }\left({\hat{\tau }}_{k},{\hat{f}}_{d}\right)=\overset{N-1}{\underset{n=0}{\sum }}s\left[nT_{s}]\cdot c[nT_{s}-{\hat{\tau }}_{k}T_{s}\right]\cdot e^{-j2\pi ({\hat{f}}_{d_{k}}+{\hat{f}}_{\delta })nT_{s}}\;$$

(11) $$\;S_{\delta }\left({\hat{\tau }}_{k},{\hat{f}}_{d}\right)=N\cdot A_{s}\cdot R\left(\Delta \tau_{k}\right)\cdot \sin c\left(\Delta f_{\delta }T_{coh}\right)\cdot e^{j\phi }+{\tilde{w}}^{\prime }\;$$

where N is the number of samples of the input signal s[·] to be coherently processed, $A_{s}$ is the signal amplitude, $R\left(\Delta \tau_{k}\right)$ is the autocorrelation function of the signal spreading code evaluated at the code phase offset $\Delta \tau_{k}$ ($\Delta \tau_{k}=\tau_{k}-{\hat{\tau }}_{k}$), $\Delta f_{d_{k}}$ is the offset between the true and candidate carrier frequencies ($\Delta f_{d_{k}}=f_{d_{k}}-{\hat{f}}_{d_{k}}$), $T_{coh}$ is the coherent integration time and $\tilde{w}$ is the resulting noise component. Thus, the new individual detection and the new CD metrics are respectively:

(12) $$\;D_{ind}\left({\hat{\tau }}_{k},{\hat{f}}_{d_{k}}\right)={\left|S_{\delta }\left({\hat{\tau }}_{k},{\hat{f}}_{d}\right)\right|}^{2}\;$$

(13) $$\;D_{\mathrm{CD}}\left(R_{i},\theta_{j},\Delta B_{n},\beta \right)=\underset{k}{\sum }D_{ind}\left({\hat{\tau }}_{k},{\hat{f}}_{d_{k}}\right)\;$$

where $D_{\mathrm{CD}}$ is calculated by the sum of the correlations of all visible satellites. 

## 3. Smart Collaborative Positioning Using CD Approach

### 3.1. Influence of Some Parameters in CD Process

**Figure 3:**
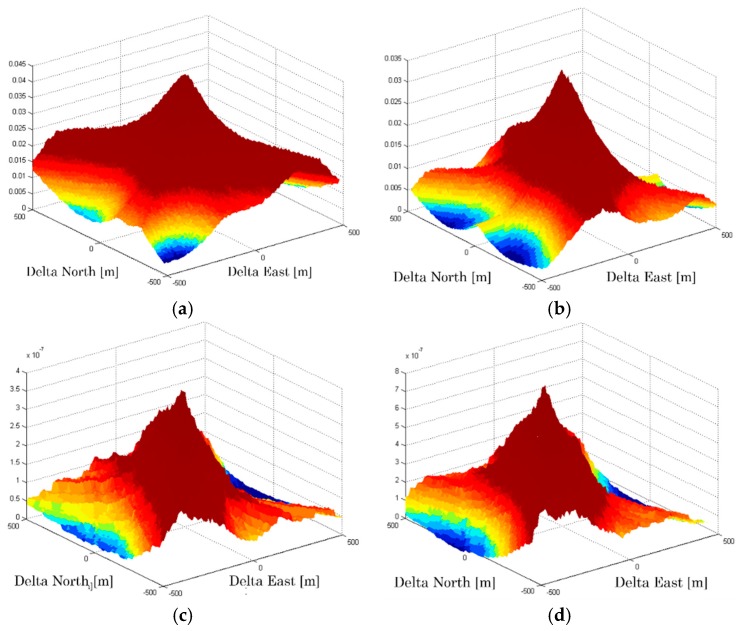

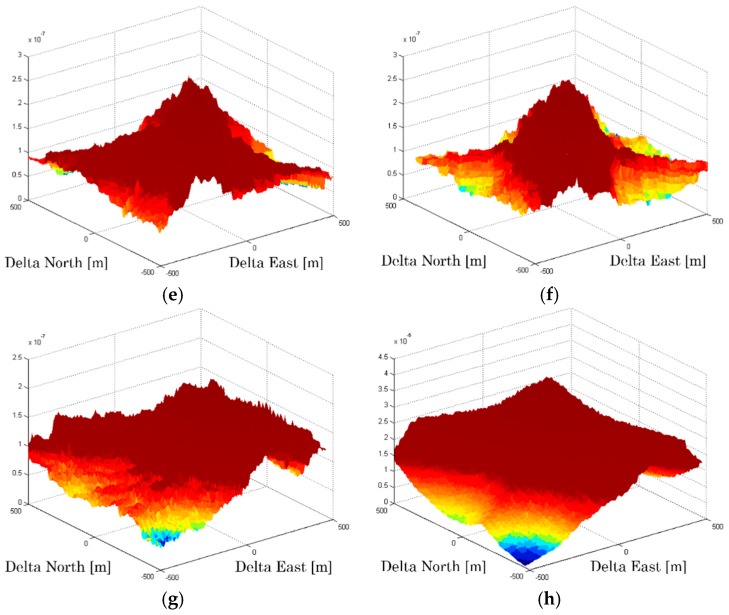
Influence on CD positioning ambiguity. (**a**) 6 satellites, mask = 10°, GDOP = 1; (**b**) 10 satellites, mask = 10°, GDOP = 1; (**c**) 6 satellites, mask = 10°, GDOP = 2.5; (**d**) 10 satellites, mask = 10°, GDOP = 2.5; (**e**) 6 satellites, mask = 30°, GDOP = 2.5; (**f**) 10 satellites, mask = 30°, GDOP = 2.5; (**g**) 6 satellites, mask = 30°, GDOP = 10; (**h**) 10 satellites, mask = 30°, GDOP = 10.

The goal of this paper is to propose a smart version of the CD approach. The developed algorithm will be able to operate the receiver optimally according to various parameters. For example, choosing the best satellites or selecting of the best reference station to assist the user. The influence of some parameters on the CD positioning ambiguity is shown in Figure 3. In this example, we compare the 3D correlogram by calculating the CD metrics corresponding to 6 best satellites and all visible satellites (10 satellites) by varying some simulated parameters ($C/N_{0}$, elevation mask angle, GDOP). It is based on the presence of three strong signals, and the remaining satellites are too weak to justify the use of the CD approach instead of the conventional acquisition. So, we have three weak + three strong for six best satellites and seven weak + three strong for 10 satellites. Weak signals correspond to the strongest signals below the set threshold, and strong signals correspond to the strongest signals among all visible satellites.

According to the curves in Figure 3, we can see that the positioning ambiguity varies according to changes in certain parameters. For example, the position solution becomes less accurate when the mask angle increases. Similarly, the position error becomes greater when the geometrical configuration is poor. Moreover, it should be noted that, for the same parameters, the curves corresponding to the 6 best satellites and 10 satellites are approximately the same, i.e., they do not present a great difference. 

### 3.2. Best Satellites Selection Algorithm in CD Process

In the proposed algorithm, the reference station can be fixed or mobile. The idea is to expand the availability of assistance for the mobile user in GNSS-challenging environments. It has been demonstrated in [5] that the mobility of the RS in the CD approach depends mainly on the integration period and the dynamic of the reference station. 

**Figure 4:**
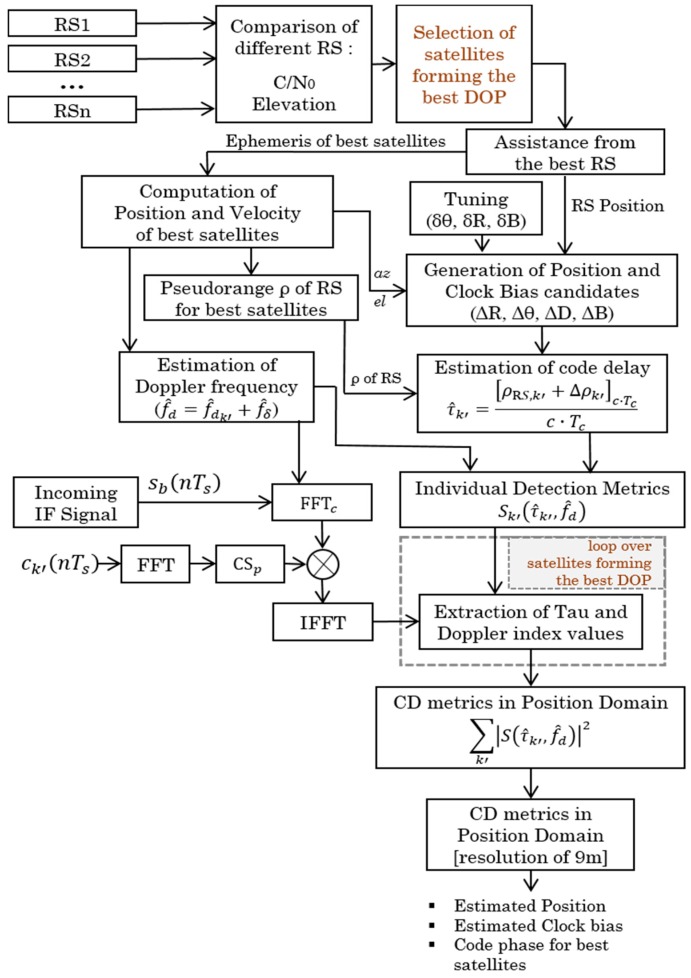
Flow chart of proposed smart cooperative positioning using CD.

Thus, in the algorithm of best satellites selection, the receiver that is in good reception condition sends its data (position, pseudorange, ephemerides) to the other receivers in difficulty to allow them to estimate their positions. Weighting costs will then be allocated to all visible satellites according to the quality of the parameters received. This will make the optimal choice of the best receiver among all the reference stations while choosing the receiver that receives the best satellites. The CD algorithm will be able to choose the best satellites intelligently. Finally, to calculate the position, the CD metric will be calculated as a function of time and Doppler. More specifically, the direct positioning algorithm is used to estimate the position according to the best selected satellites, since in the case of a conventional CD, all the available satellites are used. In fact, the algorithm consists of the optimal use of the best satellites to take advantage of the CD’s ability to increase sensitivity with fewer satellites and at the same time reduce the complexity of the operation as shown in Figure 4. To implement this idea, the CD algorithm is based on several parameters, the most important of which are the $C/N_{0}$ level, the elevation angle and the satellites forming the best DOP. The CD acquisition is performed using the BPS (Bi-dimensional Parallel Search) acquisition method to accelerate the computation during the search process in both dimensions (code and Doppler) in parallel. 

Thus, the new CD metric with the best satellites and reference station is expressed as:

(14) $$\;D_{\mathrm{CD}}\left(R_{i},\theta_{j},\Delta B_{n},\beta \right)=\underset{k_{bs}}{\sum }D_{ind}\left({\hat{\tau }}_{k},{\hat{f}}_{d_{k}}\right)\;$$

where $D_{\mathrm{CD}}$ is calculated by the sum of the correlations of all satellites selected by the algorithm, which are considered the best (satellites with higher $C/N_{0}$ and with higher elevation angles, and in some cases satellites forming the best GDOP), but not all visible satellites. That is why we have the $k_{bs}$ index with the sum operator which refers to the best satellites. 

Briefly, the smart CD algorithm involves an optimization method allowing the use of the best satellites as follows:

(1) For each RS that can assist the user:
  (i) Get the elevations and azimuths of all satellites in view and sort them with their elevations in ascending order.
  (ii) Select the better satellites with higher $C/N_{0}$:
    It is necessary to choose a certain value as a threshold for the selection of strong and weak satellites. For example, we can choose the nominal value of 45 dB-Hz as the threshold value. This can be changed according to the objectives set for the receiver design. This operation corresponds to Equation (18).
  (iii) Select the best satellites with higher elevation angles:
    It is necessary to choose a certain value as a threshold for the selection of satellites as good or bad according to their angle of elevation. To have good satellites, the threshold can be set at $10^{{}^{\circ}}$, i.e., exclude satellites with elevation lower than $10^{{}^{\circ}}$, even it is very common to define an elevation mask of $5^{{}^{\circ}}$. The algorithm will just choose the satellites having the best angles, i.e., which have the highest angles. This operation corresponds to Equation (19).
(2) Assign weights to each satellite in view corresponding to both parameters, $z_{1,k}$ for $C/N_{0}$ and $z_{2,k}$ for the elevation angle.
(3) Compute the cost function according to assigned weights and select the best satellites. If the satellites selected by each RS have the same parameters by calculating the cost function, then select the satellites that make up the best GDOP among the satellites, i.e., look up the optimal geometry according to the number of available satellites. This operation corresponds to Equation (20).
(4) Choose the best RS, which has optimal results, among the available RS which can assist the MS. The choice of the best RS can be made by comparing their statistical characteristics, i.e., the results obtained in code phase estimation from both RS. For each RS, the mean error and the standard deviation of the difference between the estimated code phase and the true code phase are compared. If all RS have the same parameters or the same costs (almost impossible in reality), choose the nearest reference station, and in the case where the RSs are at an equal distance from the MS, choose one RS randomly. Otherwise, an interesting alternative is to use two or more RS at the same time in the calculation of individual and collective detection metrics to estimate the position of the MS. The feasibility of this technique is demonstrated in [14].
(5) Send assistance data from the best RS or from both RS according to step 4.
(6) Perform CD process:
  - Correlation as a function of code phase and Doppler estimated by SPL method with delta correction, refered to Equation (16).
  - Based on polar coordinates in 2 or 3 iterations
  - CD metric is calculated as the sum of the best satellites chosen in steps 1, 2 and 3, while combining measurements from all RS.
(7) Estimate the user MS position.

To select the best RS having the best parameters, the decision-making process is multi-objective oriented where the cost functions help making the decision and it is modeled as:

(15) $$\;\max\left\{y\right\}=f\left(\bar{z}\right)=\left[f\left({\bar{z}}_{1,k}\right),f\left({\bar{z}}_{2,k}\right)\right]\;$$

where ${\bar{z}}_{1,k}=\left(z_{1,1},z_{1,2},\ldots ,z_{1,k}\right)\in Z_{1}$ corresponds to the $C/N_{0}$ for all satellites in view; ${\bar{z}}_{2,k}=\left(z_{2,1},z_{2,2},\ldots ,z_{2,k}\right)\in Z_{2}$ corresponds to the elevation angle for all visible satellites; and $\bar{y}=\left(y_{1},y_{2},\ldots ,y_{n}\right)\in Y$ corresponds to steps 3 and 4 of the above procedure.

In case we need to use more than one reference station, we can merge the measurements obtained from the different RS and then calculate the metrics for individual as follows [14]:

(16) $$\;D_{ind}\left(R_{i},\theta_{j},\Delta B_{n}\right)=\overset{Rn}{\underset{n=1}{\sum }}w_{n}{\left|S_{\delta }\left({\hat{\tau }}_{k}^{n},{\hat{f}}_{d_{k}}^{n}\right)\right|}^{2}\;$$

where n represents the reference station number, $R_{n}$ represents the total number of reference stations, and $w_{n}$ represents the weight of each RS corresponding to its parameters, such as:

(17) $$\;w_{n}\left[{\left(\;C/N_{0}\right)}_{k},el_{k}\right]=w_{n,1}\left[{\left(C/N_{0}\right)}_{k}\right]w_{n,2}\left(el_{k}\right)\;$$

For $C/N_{0}$, weighting cost is calculated as:

(18) $$\;w_{n,1}\left(x\right)=\{\begin{array}{c} 10^{\frac{x-45}{\alpha }}{\left(\left(V_{f}\times 10^{\frac{P-45}{\alpha }}-1\right)\frac{x-45}{P-45}+1\right)}^{-1},\;\;x<45\;\mathrm{dBHz}\\ 1\;\;\;\;\;\;\;\;\;\;\;\;\;\;\;\;\;\;\;\;\;\;\;\;\;\;\;\;\;\;\;\;\;\;\;\;\;\;\;\;\;\;\;\;\;\;\;\;\;\;\;\;\;\;\;\;\;\;\;\;\;\;\;\;\;\;\;,\;\;x\geq 45\;\mathrm{dBHz} \end{array}\;$$

where x represents the value of $C/N_{0}$ (dB-Hz), α is a parameter to adjust the curve of the weighting function $w_{n}\left(C/N_{0}\right)$ as defined in [15], $V_{f}$ controls the value of the weighting function $w_{n}$ for x=P, i.e., $w_{n}\left(P\right)=\frac{1}{V_{f}}$, P is the value of $C/N_{0}$ for which the weighting function $w_{n}$ is forced in order to obtain the weight defined by parameter $V_{f}$, and γ is the threshold value is defined (step 1.i. of the algorithm description). According to [15], the optimal values obtained for the different parameters of the function $w_{n,1}$ are: $\left(\alpha ,\;V_{f},\;P\right)=\left(80,\;30,\;20\right)$.

The second criterion is the satellite selection method based on maximal elevation angle during the observation period. For the elevation angle parameter, the weighting function is defined as [15]:

(19) $$\;w_{n,2}\left(x\right)=\{\begin{array}{c} \;\frac{\sin^{2}\left(x\right)}{\sin^{2}\left(10^{{}^{\circ}}\right)},\;\;x<10^{{}^{\circ}}\\ 1\;\;\;\;\;\;\;\;\;\;\;\;\;,\;\;x\geq 10^{{}^{\circ}} \end{array}\;$$

According to step 3 in the best satellite selection algorithm, if the satellites selected by each RS have the same parameters by calculating the cost function, then select the satellites that make up the best GDOP among the selected satellites as:

(20)  min{GDOP(x)}≤6 

Finally, the collective detection metric can be expressed in function of the best satellites as:

(21) $$\;D_{\mathrm{CD}}\left(R_{i},\theta_{j},\Delta B_{n}\right)=\underset{k_{bs}}{\sum }D_{ind}\left({\hat{\tau }}_{k_{bs}}^{n},{\hat{f}}_{d_{k_{bs}}}^{n}\right)=\underset{k_{bs}}{\sum }\left\{\overset{Rx}{\underset{n=1}{\sum }}w_{n}{\left|S_{\delta }\left({\hat{\tau }}_{k_{bs}}^{n},{\hat{f}}_{d_{k_{bs}}}^{n}\right)\right|}^{2}\right\}\;$$

where $k_{bs}$ represents the number of the best satellites, and ${\hat{\tau }}_{k_{bs}}^{n}$ represent the estimated code delay corresponding to the best satellites $k_{bs}$ for a hypothetical location $\left(R_{i},\theta_{j},\Delta D_{m},\Delta B_{l}\right)$ w.r.t each RS as:

(22) $$\;{\hat{\tau }}_{k_{bs}}^{1}=\frac{{\left[\rho_{\mathrm{RS}1,k_{bs}}+\Delta \rho_{k_{bs},1}\left(R_{i},\theta_{j},\Delta D_{m},\Delta B_{l}\right)\right]}_{c\cdot T_{code}}}{c\cdot T_{code}}\cdot N_{code}\;\;{\hat{\tau }}_{k_{bs}}^{2}=\frac{{\left[\rho_{\mathrm{RS}2,k_{bs}}+\Delta \rho_{k_{bs},2}\left(R_{i},\theta_{j},\Delta D_{m},\Delta B_{l}\right)\right]}_{c\cdot T_{code}}}{c\cdot T_{code}}\cdot N_{code}\;\ldots \;\;{\hat{\tau }}_{k}^{n}=\frac{{\left[\rho_{\mathrm{RS}n,k}+\Delta \rho_{k,n}\left(R_{i},\theta_{j},\Delta D_{m},\Delta B_{l}\right)\right]}_{c\cdot T_{code}}}{c\cdot T_{code}}\cdot N_{code}\;$$

where $\Delta \rho_{k_{bs},1}$ and $\Delta \rho_{k_{bs},2}$ are the difference in pseudorange from the MS w.r.t the one measured by the RS1 and RS2, respectively, for the best satellites $k_{bs}$, as:

(23) $$\;\Delta \rho_{k_{bs},1}=-R_{1}\cos\left(el_{k_{bs},1}\right)\cos\left(az_{k_{bs},1}-\theta_{1}\right)+c\cdot \Delta B_{1}\;\;\Delta \rho_{k_{bs},2}=-R_{2}\cos\left(el_{k_{bs},2}\right)\cos\left(az_{k_{bs},2}-\theta_{2}\right)+c\cdot \Delta B_{2}\;\ldots \;\;\Delta \rho_{k_{bs},n}=-R_{n}\cos\left(el_{k_{bs},n}\right)\cos\left(az_{k_{bs},n}-\theta_{n}\right)+c\cdot \Delta B_{n}\;$$

Thus, the acquisition problem is to search for the optimal vector X=(R,θ,ΔB) in the search space R∈[0 m, 10,000 m], θ∈[0°, 360°], and ΔB∈[−150,000 m, 150,000 m] which maximizes the criterion function:

(24) $$\;J=\mathrm{fun}\left(D_{\mathrm{CD}}\left(R_{i},\theta_{j},\Delta B_{l}\right)\right)\;\;=\mathrm{fun}\left(\underset{k_{bs}}{\sum }D_{ind}\left({\hat{\tau }}_{k_{bs}}^{n},{\hat{f}}_{d_{k_{bs}}}^{n}\right)\right)\;\;=\;\mathrm{fun}\left(\underset{k_{bs}}{\sum }\overset{R_{n}}{\underset{n=1}{\sum }}w_{n}{\left|S_{\delta }\left({\hat{\tau }}_{k_{bs}}^{n},{\hat{f}}_{d_{k_{bs}}}^{n}\right)\right|}^{2}\right)\;$$

where “fun” is an increasing function in which the decision of detection is made with the surpass of a threshold determined by a pre-defined false-alarm probability.

## 4. Experimental Results and Performance Analysis

To practically demonstrate the efficiency of this application in selecting the best satellites, tests with real GPS L1 C/A were carried out. Two uBlox LEA-6T were used as RS1 and RS2 to collect series of measurements in order to assist the MS. Both reference receivers were in good condition and set to receive satellite signals at spots with open sky. RS1 was placed in a parking lot (N 45°49′64.29″, W 73°56′42.78″, 83.61 m), and RS2 in the middle of a green park (N 45°49′17.66″, W 73°56′16.07″, 83.59 m). A bit grabber USRP B210 was used to collect raw data as MS in a narrow street in downtown Montreal (N 45°29′34.8″, W 73°33′49.4″, 83.60 m) where acquiring satellite signals is generally difficult due to concentrated building structures. 

**Figure 5:**
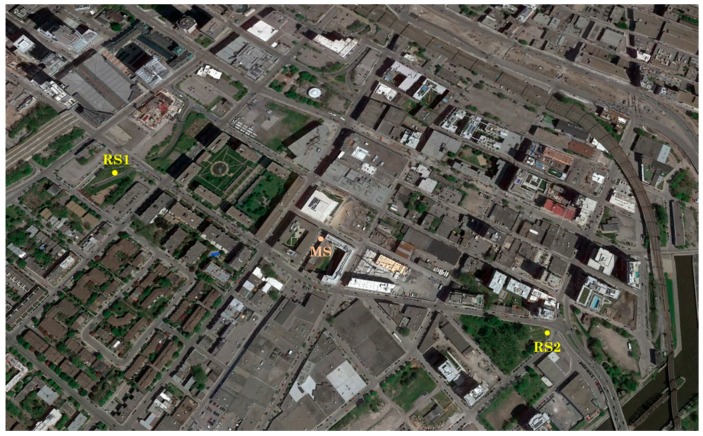
Position of MS and both RSs during satellite signals recording in downtown Montreal.

**Figure 6:**
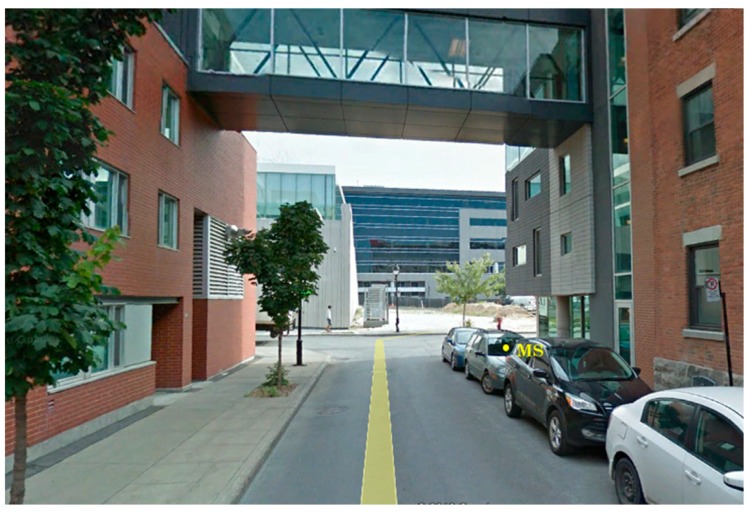
Location of MS in Montreal downtown (GPS antenna placed inside a car).

The position of the two reference receivers (RS1 and RS2) that assist the MS in downtown Montreal is shown in Figure 5 and they were in a good sky visibility. Figure 6 shows the location of the MS raw data acquisition where the GPS L1 C/A signals are degraded because of the high buildings around and poor sky conditions. 

The measurement campaign was carried out for 4 h and the values are taken every 1 h. During this observation period, the data received changes ($C/N_{0}$ level, elevation angle, forming GDOP) as time progresses. The received satellites during the entire observation period by each receiver are summarized in Table 1 ($C/N_{0}$ level in (dB-Hz) and elevation angle in (°)). Note that the real distances between the receivers are: RS1–RS2 = 816.46 m, RS1–MS = 412.03 m, and RS2–MS = 424.66 m. 

The horizontal uncertainty range was set to 20 km to reflect a realistic application scenario as shown in Table 2. Collective acquisition was performed by three iterations, with the search space refined at each iteration until an accurate estimate of the MS position could be obtained as we can be seen in Table 2.

### 4.1. Smart Collaborative Positioning: Selection of the Best RS

In the practical case, the measurements of RS1 and RS2 are almost identical as we observe in Table 1. In order to analyze the performance of the algorithm of the best RS selection and the best satellites selection, we will still focus on the key parameters ($C/N_{0}$ and elevation angle) received by both RS and MS.

The idea is that the CD algorithm is able to choose the best RS using the best satellites to optimize the CD process while reducing complexity with fewer satellites. Two criteria are used to choose which of RS1 and RS2 will be used to assist MS: $C/N_{0}$ and elevation angle parameters. 

In Equations (20) and (21), the thresholds used for $C/N_{0}$ and elevation angle are respectively 45 dB-Hz and $10^{{}^{\circ}}$. From Table 1, 11 satellites are received by RS1 and RS2 (SV 1, SV 3, SV 6, SV 11, SV 17, SV 18, SV 19, SV 22, SV 24, SV 28, SV 30) and nine satellites are received by MS (SV 1, SV 3, SV 6, SV 11, SV 17, SV 19, SV 22, SV 28, SV 30) at the end of the recording.

On the 11 visible satellites, seven satellites received by RS1 (green part) are stronger than those received by RS2 (SV 1, SV 6, SV 11, SV 17, SV 18, SV 22, SV 28), one satellite received by RS1 (orange part) is weaker than those received by RS2 (SV 30), and the remainder have the same $C/N_{0}$ level. This means that the satellites received by RS1 are stronger than those received by RS2 in terms of $C/N_{0}$. Then, among these seven strong satellites received by RS1, there are six satellites which have elevation angles lower than those of RS2 (SV 1, SV 6, SV 11, SV 17, SV 22, SV 28). In order to choose the best RS to be used to help the MS to roughly estimate its position, we have compared the statistical characteristics of results obtained in code phase estimation from both RS. For each RS, the mean error and the standard deviation of the difference between the estimated code phase and the true code phase are compared. This leads to the logical choice of RS1 as an assistant to MS in the smart CD algorithm. 

In this case, since we have been able to distinguish the satellites to be chosen according to the key parameters, we do not need to see which satellites form the best GDOP among those chosen. In addition, here we have a single satellite at more than 45 dB-Hz (strong) in view of the MS and the remains are considered weak satellites. Hence, based on the principle of CD, the algorithm will not be limited to the minimum of satellites, but will extend to use the best possible satellites.

To properly analyze the impact of using the best satellites or the best RS in the CD algorithm, compare the following four functions and evaluate the performance in terms of sensitivity and complexity:

(1) CD in function of best satellites and best RS “CD/Best_Sat/Best_RS”,
(2) CD in function of best satellites and all RS “CD/Best_Sat/All_RS”,
(3) CD in function of all visible satellites and best RS “CD/All_Sat/Best_RS”, and
(4) CD in function of all visible satellites and all RS “CD/All_Sat/All_RS”.

For the CD process using the best RS and/or the best satellites, the algorithm described in Section 3.2 is used. In cases where all RS are used, the CD algorithm described in Equation (23) is used, in which the measurements of RS1 and RS2 are combined in the CD algorithm. The best RS is RS1, the best satellites are: SV 1, SV 6, SV 11, SV 17, SV 22, SV 28; and all satellites are: SV 1, SV 3, SV 6, SV 11, SV 17, SV 19, SV 22, SV 28, SV 30. 

### 4.2. Sensitivity Analysis

**Figure 7:**
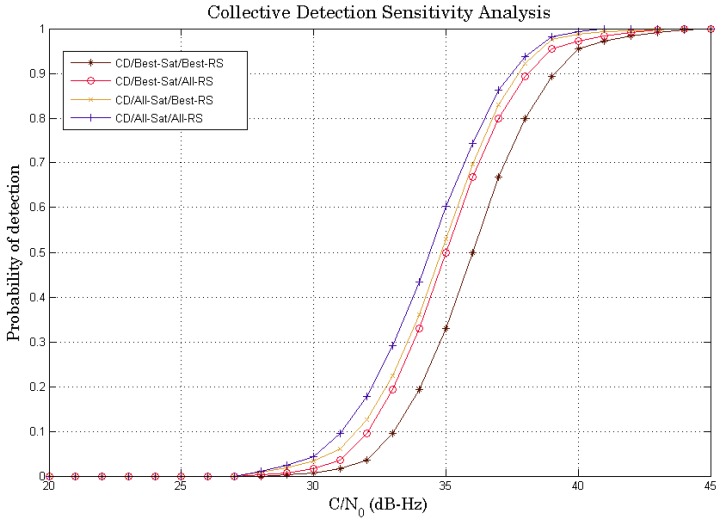
Sensitivity performance comparison between all four CD algorithms.

SPL with delta correction technique is used for Doppler estimation in all 4 CD algorithms described previously. Knowing that, in the basic idea of the CD approach, the CD is advantageous if we have as many satellites as possible, but we will compare if there is a big difference between the operation of the best satellites and the exploitation of all available satellites. Considering the previous analysis, the algorithm will compute the CD metric using six satellites (best satellites) instead of nine satellites (all visible satellites). Figure 7 shows the comparison of the sensitivity analysis curves of all four CD algorithms: “CD/Best_Sat/Best_RS” (six satellites/RS1), “CD/Best_Sat/All_RS” (six satellites/RS1–RS2), “CD/All_Sat/Best_RS” (nine satellites/RS1) and “CD/All_Sat/All_RS” (nine satellites/RS1–RS2).

It can be seen that the “CD/All_Sat/All_RS” algorithm is the best algorithm in terms of sensitivity. The idea of combining all available satellites is the basic principle of the CD approach to increase sensitivity as much as possible. The algorithms of “CD/Best_Sat/All_RS” and “CD/All_Sat/Best_RS” have almost the same performance. “CD/Best_Sat/Best_RS” has the lowest performance among all the algorithms (−2 dB-Hz compared to “CD/All_Sat/All_RS”). This is due to the use of some selected satellites instead of all available satellites. On the other hand, the difference between “CD/All_Sat/All_RS” and “CD/Best_Sat/All_RS” is not very remarkable, which could be interesting if we look at the compromise between sensitivity (±0.7 dB-Hz) and complexity (nine satellites vs. six satellites). Thus, using the best satellites makes it possible to have a reasonable sensitivity performance whereas there is a gain in complexity since there is less computation, which makes this proposed strategy interesting. 

**Figure 8:**
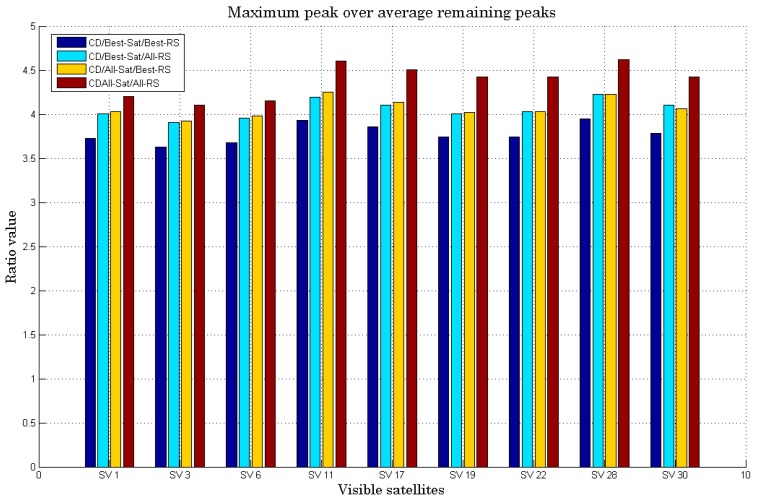
Ratio of maximum peak/average of remaining peaks of all 4 CD algorithms.

To better analyze the sensitivity performance of the all CD algorithms and to demonstrate these discussions, it is also important to see the ratio between the maximum peak and the average of the remaining peaks for each detected satellite. It is shown in Figure 8. In fact, the maximum peak is the peak corresponding to the best combination of code/Doppler during the correlation for each satellite in view. The histograms show the peak of detection corresponding to each visible satellite. For each satellite, we calculate the value of the maximum correlation peak divided by the average value of all the remaining peaks to better analyze the sensitivity.

The histograms in Figure 8 show the better performance using all the satellites (the basic idea of CD) and combine the two RS compared to other algorithms. We can also notice that the algorithms of “CD/Best_Sat/All_RS” and “CD/All_Sat/Best_RS” have almost the same performance. We can even see that “CD/Best_Sat/All_RS” is better than “CD/All_Sat/Best_RS” for SV 30, which may be due to the fact that RS2 receives a higher signal power than RS1 even if the difference is smaller. To better choose the right algorithm, complexity is a very important performance metric that needs to be studied.

### 4.3. Complexity Analysis

According to the CD process parameters in Table 2, there are 42,142 candidate points in the execution of the “CD/All_Sat/All_RS” algorithm. The CD algorithm takes 2880 ms to execute the acquisition process of 20 code periods to process all satellites in view (nine satellites) and combining the RS1 and RS2 measurements whereas 1860 ms to process all satellites in view with only RS1 measurement (best RS). For “CD/Best_Sat/All_RS” algorithm, the execution time takes 1920 ms whereas 1240 ms if RS1 only is used. We can see that the execution of “CD/Best_Sat/All_RS” algorithm is 1.5 times faster than “CD/All_Sat/All_RS”. Table 3 shows some overall conclusion of the performance of algorithms based on the criteria used.

**Figure 9:**
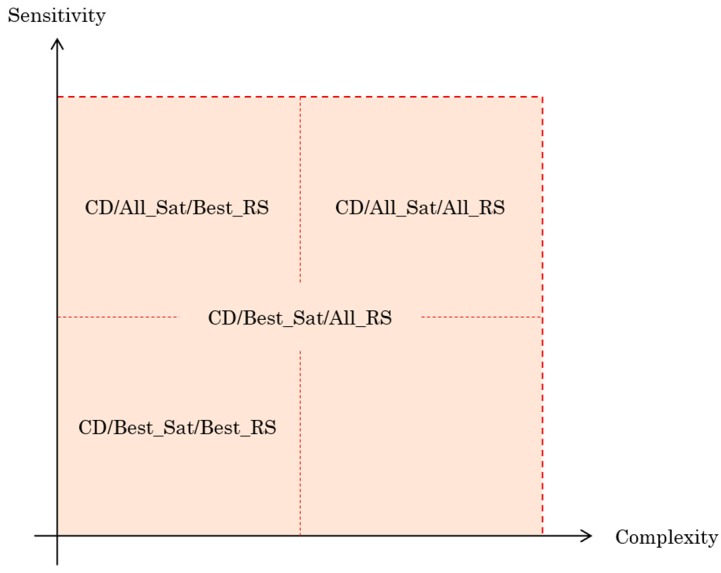
Compromise Sensitivity-Complexity of all 4 CD algorithms.

To better see the tradeoff between these two important performance metrics, Figure 9 shows the 4 CD algorithms compared in this study. The choice to use all visible satellites or to select the best ones as well as all the reference stations or the best one depends on the desired application. Here we can choose to use the best satellites while combining all reference stations that meets the criterion “not very complex but allows to have a good sensitivity”.

### 4.4. Accuracy Analysis: Working with Optimum Number of Parameters

**Figure 10:**
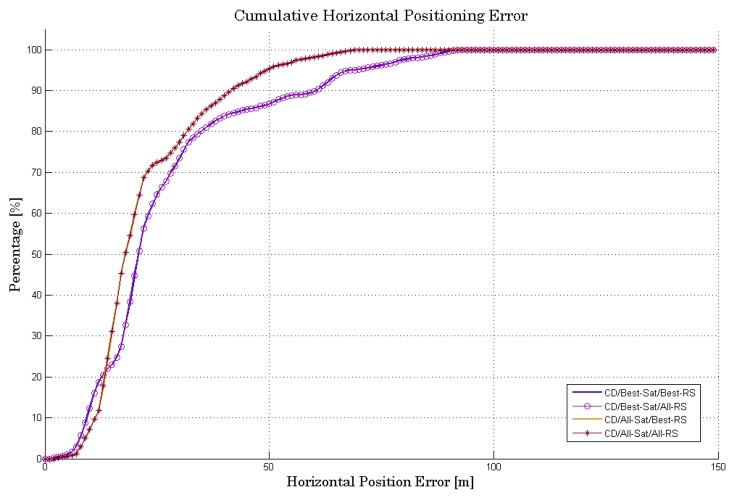
Comparison of HPE between the four CD algorithms.

Apart from the complexity and sensitivity, the performance of the four CD algorithms in terms of accuracy has to be analyzed. To illustrate the benefit of using best satellites instead of all satellites in view (complexity vs. sensitivity), let’s compare the cumulative horizontal positioning error (HPE) of each CD algorithm, as shown in Figure 10. 

By observing these curves, we can see that the fact of combining two reference stations in the CD process has no impact on accuracy. We can see that the CD algorithm using all visible satellites has less positioning error than the others. Despite this, there is no significant difference between the HPE using only six best satellites and nine satellites (all satellites in view). This can be explained by the low influence of the weak signals exploitation, which come to collaborate to the collective detection metrics. We can deduce from this that we can limit the processing to only 6 satellites in order to minimize the computational load, with the goal being to use the minimum possible number of satellites while retaining a precise positional solution. The motivation idea is to carry out the minimum of calculations while retaining a good margin of precision of position. 

## 5. Conclusions

In order to achieve the objectives of GNSS receiver design (availability, integrity, accuracy and electromagnetic vulnerability), the smart module proposed in this work allows the optimal selection of the best signal processing technique to use. The idea is to allow the receiver to process the received signal according to certain parameters: number of visible satellites, received signal level, elevation angle, geometric configuration of available satellites, navigation environment, etc. This module contains only the intelligent algorithm for selecting the best technique to use, namely, conventional HS-GNSS acquisition method or CD approach, while exploiting the best reference station for assistance and processing the best satellites.

In this paper, the CD algorithm is optimized with a smart selection of the best satellites in the direct positioning technique instead of all visible satellites, in order to reduce the number of required computations. 

From the different obtained results, we can deduce that using the best satellites while combining all reference stations measurements may be sufficient to process the position solution instead of performing a process on all visible satellites. It can reduce the required computational burden (1.5 time faster) and at the same time have a good receiver sensitivity (loss of ±0.7 dB-Hz), and also stay in a good margin of positioning accuracy. Indeed, this can reduce the complexity of implementation, but especially, it can minimize the energy consumption as part of a multi-GNSS receiver where tens of signals are present in the receiver antenna. 

The choice of the assigned weight costs may depend on several criteria on the quality of the corresponding reference receiver (low cost or professional receivers, acceptable margin of error, receivers that know their position with very high precision, etc.). In the practical case, the choice of the right technique will depend on the availability of the good receivers.

The strategy of CD application as smart cooperative positioning meets the requirements of the complexity-sensitivity trade-off in CD approach. This concept allows us to take advantage from the existence of several receivers in good condition to help a receiver in deep urban area to estimate its position where it is usually impossible. Internet of Things (IoT) technology will also accelerate this possibility in the future.

Future work for this research will focus on real-time testing with some receivers to verify the practical feasibility of this application. The assistance data update, taking into account the change of reference receiver, according to the evolution of the MS-RS distance should also be studied. 

## Figures and Tables

**Table 1:** Visible satellites during observation period.

Rx	14 H		15 H		16 H		17 H		18 H		$\mathrm{Mean}\;C/N_{0}\;$	Mean Elevation
	SV	El	SV	El	SV	El	SV	El	SV	El		
RS1			1	37.01	1	59.36	1	61.03	1	43.21	45.75	50.15
							3	20.71	3	35.79	37.75	28.25
									6	26.51	37.00	26.51
	7	55.34	7	78.33	7	59.48	7	21.31			46.25	53.61
	8	86.12	8	60.90	8	42.19	8	18.29			46.00	51.87
	9	37.01									43.25	37.01
	11	28.98	11	63.24	11	76.47	11	51.46	11	33.47	46.00	50.72
			13	12.57	13	16.75					34.25	14.66
	16	26.87									39.75	26.87
					17	18.20	17	43.12	17	56.94	43.75	39.42
	18	21.34	18	24.99	18	58.91	18	39.19	18	21.01	40.50	33.08
							19	23.54	19	39.03	38.50	31.28
							22	27.62	22	32.46	38.50	30.04
	23	17.57									33.50	17.57
									24	12.59	34.00	12.59
	27	51.97	27	24.34							42.75	38.15
	28	12.79	28	34.08	28	49.03	28	74.28	28	80.08	46.00	50.05
	30	30.98	30	57.64	30	67.24	30	44.17	30	21.31	43.50	44.26
RS2			1	36.94	1	59.16	1	61.01	1	43.03	45.50	50.03
							3	20.64	3	35.88	37.75	28.26
									6	26.03	36.75	26.03
	7	55.32	7	78.17	7	59.27	7	21.17			46.25	53.48
	8	86.09	8	60.54	8	42.01	8	18.09			46.00	51.68
	9	36.97									43.25	36.97
	11	28.95	11	63.20	11	76.36	11	51.27	11	33.21	45.50	50.59
			13	12.34	13	16.49					34.00	14.41
	16	26.72									39.75	26.72
					17	18.07	17	43.05	17	56.18	43.50	39.10
	18	21.14	18	24.87	18	58.89	18	39.09	18	21.17	40.00	33.03
							19	23.47	19	39.01	38.50	31.24
							22	27.59	22	32.29	38.25	29.94
	23	17.37									33.25	17.37
									24	12.33	34.00	12.33
	27	51.67	27	24.26							42.75	37.96
	28	12.42	28	34.01	28	49.00	28	74.23	28	79.78	45.75	49.88
	30	30.69	30	57.39	30	67.21	30	44.15	30	21.10	43.75	44.10
MS			1	36.95	1	58.99	1	60.85	1	43.02	45.75	49.95
									3	34.78	37.50	34.78
									6	25.89	36.75	25.89
	7	54.94	7	77.69	7	58.88	7	21.02			41.25	53.13
	8	86.02	8	60.23	8	41.76	8	17.79			39.00	51.45
	11	28.75	11	62.95	11	76.12	11	51.06	11	32.87	37.50	50.35
					17	18.00	17	42.84	17	56.43	41.75	39.09
							19	23.17	19	38.87	37.50	31.02
							22	27.33	22	32.22	36.25	29.77
	27	51.63	27	23.77							43.75	37.70
	28	12.40	28	33.68	28	48.27	28	73.87	28	79.74	42.75	49.59
	30	30.21	30	57.10	30	66.59	30	43.75	30	21.02	39.75	43.73

**Table 2:** Parameters of CD process.

Item		Rough 1st Iteration	Medium 2nd Iteration	Fine 3rd Iteration
Horizontal dimension	Radial Uncertainty [m]	±10,000	±2922	±292
	Radial Step Size [m]	2922	292	29.2
	Angular Step size [°]	14.4	5.7	5.7
Clock Bias	Clock Bias Uncertainty [m]	±150,000	±220	±22
	Clock Bias Step Size [m]	440	44	4.4

**Table 3:** Parameters of CD process.

	RS	Best RS	All RS
Satellite			
Best Satellites		less sensitive less complex	less sensitive more complex
All Satellites		more sensitive less complex	more sensitive more complex

## References

[1] Seco-Granados G., López-Salcedo J., Jiménez-Baños D., López-Risueño G. (2012). Challenges in Indoor Global Navigation Satellite Systems: Unveiling its Core features in Signal Processing. IEEE Signal Process. Mag..

[2] DiEsposti R. (2007). GPS SV Code Signal Processing and Receiver Design for Simultaneous All-In-View Coherent Signal Acquisition and Navigation Solution Determination.

[3] Axelrad P., Bradley B., Donna J., Mitchell M., Mohiuddin S. (2011). Collective detection and direct positioning using multiple GNSS satellites. J. Inst. Navig..

[4] Cheong J. (2012). Signal Processing and Collective Detection for Locata Positioning System. Ph.D. Thesis.

[5] Andrianarison M., Sahmoudi M., Landry R. Cooperative Detection of Multiple GNSS Satellite Signals in GNSS-Challenged Environments. Proceedings of the 28th ITM of ION, ION GNSS+ 2015.

[6] Omar A.B., Sahmoudi M., Esteves P., Ries L., Andrianarison M., Landry R. (2014). A New Method of Collective Acquisition of Multiple GNSS Satellite Signals in Challenging Environments.

[7] Esteves P., Sahmoudi M., Ries L. (2014). Collective Detection of Multi-GNSS Signals: Vector-Acquisition Promises Sensitivity and Reliability Improvement. Inside GNSS Magazine.

[8] Andrianarison M., Sahmoudi M., Landry R. Innovative Techniques for Collective Detection of Multiple GNSS Signals in Challenging Environments. Proceedings of the 2016 International Conference on Indoor Positioning and Indoor Navigation (IPIN), Alcala de Henares.

[9] Li L., Cheong J., Wu J., Dempster A. (2014). Improvement to Multi-resolution Collective Detection in GNSS Receivers. J. Navig..

[10] Andrianarison M., Sahmoudi M., Landry R. (2017). Efficient and Innovative Techniques for Collective Acquisition of Weak GNSS Signals. J. Comput. Commun. Spec. Issue Navig..

[11] Esteves P. An Innovative and Efficient Frequency Estimation Method for GNSS Signals Acquisition. Proceedings of the 26th International Technical Meeting of The Satellite Division of the Institute of Navigation (ION GNSS+ 2013).

[12] He Z., Renaudin V., Petovello M., Lachapelle G. (2013). Use of High Sensitivity GNSS Receiver Doppler Measurements for Indoor Pedestrian Dead Reckoning. Sensors.

[13] Van Diggelen F. (2009). A-GPS: Assisted GPS, GNSS, and SBAS.

[14] Andrianarison M., Sahmoudi M., Landry R. (2018). New Strategy of Collaborative Acquisition for Connected GNSS Receivers in Deep Urban Environments. J. Position..

[15] Lesouple J., Barbiero F., Sahmoudi M., Tourneret J.-Y., Vigneau W. (2018). Multipath Mitigation for GNSS Positioning in Urban Environment Using Sparse Estimation. IEEE Trans. Intell. Trans. Syst..

